# Waste‐Valorized Nanowebs for Crystal Violet Removal from Water

**DOI:** 10.1002/smsc.202300286

**Published:** 2024-02-02

**Authors:** Qaisar Maqbool, Isabella Cavallini, Niusha Lasemi, Simona Sabbatini, Francesca Tittarelli, Günther Rupprechter

**Affiliations:** ^1^ Department of Materials, Environmental Sciences and Urban Planning (SIMAU) INSTM Research Unit Università Politecnica delle Marche via Brecce Bianche 12 60131 Ancona Italy; ^2^ Institute of Materials Chemistry TU Wien Getreidemarkt 9/BC A‐1060 Vienna Austria

**Keywords:** adsorption, crystal violet, electrospun nanoweb, nanocellulose, wastewater treatment

## Abstract

Lightweight, metal‐free, sustainable, and reusable adsorbent materials are of paramount significance in addressing the challenges of wastewater treatment. Accordingly, semi‐crystalline nanocellulose (NC) is extracted from tissue paper waste and used to modify polyacrylonitrile (PAN) to produce electrospun nanowebs with strand diameters from ≈180–300 nm. The incorporation of NC into PAN is confirmed by infrared and Raman spectroscopy and X‐Ray diffraction. When tested for crystal violet (CV) adsorption, NC‐modified PAN (20% NC@PAN) exhibits the highest CV removal capacity, achieving 91–94% removal over three cycles each, demonstrating exceptional recyclability. In contrast, unmodified PAN significantly decreases in CV adsorption capacity (from 59% to 48% in the third cycle), possibly due to an increased (≈36%) nanofiber diameter. The adsorption kinetics, exhibiting pseudo‐second order, interparticle (in between nanofibers) diffusion, and Elovich kinetic models emphasize the role of multilayer CV adsorption through reversible chemical interactions. Confocal micro‐Raman spectroscopy unveils a multifaceted CV adsorption mechanism, suggesting both surface and multilayer diffusion, with NC‐enhancing interactions. These findings demonstrate the potential of NC‐modified PAN nanowebs as effective and environmentally sustainable adsorbents for removing CV from aqueous solutions, suggesting promising practical applications.

## Graphical abstract

Lightweight, metal‐free, semi‐crystalline nanocellulose was extracted from tissue paper waste and used to modify polyacrylonitrile to produce electrospun nanowebs. The waste‐derived nanowebs were highly efficient adsorbents for crystal violet removal from water, suggesting promising wastewater treatment applications.

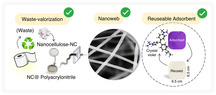

## 1 Introduction

Industrially used organic dyes are a major cause of wastewater pollution. Approximately, 700 000 tons of synthetic dyes are produced annually^[^ ^1^ ^]^ and according to World Bank Group estimates, 20% of wastewater originates from fabric dyeing and treatment processes.^[^ ^2^ ^]^ Most of the industrial dyes, such as azo dyes and their breakdown metabolites, are resistant to natural decomposition in the environment. Azo dyes are widely used as synthetic colorant in textile industry and are primarily characterized by one or more azo bonds (—N=N—). These organic pollutants are classified as xenobiotics and exhibit potential toxicity toward living organisms.^[^ ^3^, ^4^ ^]^

The dye crystal violet (CV) also represents a significant challenge due to its high water solubility.^[^ ^5^ ^]^ CV consists of aromatic rings and quaternary ammonium groups, which form strong π–π interactions and electrostatic attractions, respectively, leading to its resistance to degradation. This structural stability lets CV persist in water bodies for prolonged periods, resulting in severe environmental contamination. Exposure to CV‐contaminated water has been linked to various health issues in humans, including ocular toxicity,^[^ ^6^, ^7^ ^]^ cystitis,^[^ ^8^ ^]^ skin irritation,^[^ ^9^ ^]^ brain dysfunction,^[^ ^10^ ^]^ and potential carcinogenic effects.^[^ ^11^, ^12^, ^13^, ^14^, ^15^ ^]^

Removal of CV from wastewater is a challenging task. Various methods have been explored to tackle the removal of CV from water, each with its own set of limitations.^[^ ^3^ ^]^ Existing methods include electrochemical technologies such as electrocoagulation (EC), an advanced electrochemical method: It operates by generating metal‐OH flocs (amorphous larger clumps) within the wastewater, which can subsequently be purified through electro‐dissolution of soluble anodes.^[^ ^16^, ^17^ ^]^ Coagulation–flocculation is another water treatment process that removes dyes by adding chemicals to water. Prehydrolyzed coagulants (such as polyaluminum chloride) neutralize charged particles, and flocculants make them stick together to form larger clumps/flocs. The flocs settle at the bottom or can be filtered out, effectively clearing the water from dyes.^[^ ^18^ ^]^ Another method is biodegradation, mostly involving yeasts. The primary focus of yeast‐mediated azo dye decolorization involves the pivotal role of azo‐reductases (enzymes) in catalyzing the cleavage of azo groups (—N=N—).^[^ ^19^, ^20^ ^]^ However, these methods often fall short in achieving complete dye removal and generate harmful sludge as by‐product, posing additional disposal challenges, which are costly or require a bioreactor for cell culturing. Additionally, they may not be effective in removing trace amounts of dyes, making them unsuitable for applications requiring high purity water.

Among all, nanomaterials (NMs)^[^ ^21^, ^22^ ^]^ have emerged as promising alternatives for the removal of CV from water. Notably, NMs exhibit a high specific surface area, selectivity, and reactivity, leading to high CV adsorption capacity. Still, some NMs used as adsorbents for CV removal suffer from limitations. For instance, TiO_2_‐based NMs require artificial UV light for activation, which restricts their applicability to sunlight‐exposed regions. Similarly, graphene quantum dots,^[^ ^23^ ^]^ graphene‐oxide‐doped hydrogels,^[^ ^24^ ^]^ activated carbon,^[^ ^25^, ^26^ ^]^ biochar,^[^ ^3^, ^27^, ^28^, ^29^ ^]^ and metal–organic framework‐based NMs^[^ ^30^ ^]^ often face difficulties in achieving efficient powder dispersion because of aggregation tendency and challenging recovery through centrifugation (or filtration), limiting their practical use. Additionally, carbon nanotubes,^[^ ^31^ ^]^ despite their excellent adsorption properties,^[^ ^32^, ^33^ ^]^ may be expensive and pose potential health risks if released into the environment.^[^ ^34^, ^35^ ^]^ Furthermore, magnetic NMs, although efficient in CV adsorption,^[^ ^36^, ^37^, ^38^ ^]^ may generate metal ions or require magnetic separation processes, making them less convenient for large‐scale applications. These limitations warrant further research to explore more effective, manageable, and sustainable NMs for CV removal from water, preferably as metal‐free systems.

Apart from the various NMs discussed so far, electrospun nanofibers received considerable attention as efficient CV adsorbents.^[^ ^39^ ^]^ Electrospinning is a versatile technique that allows the production of nanofibers with a high specific surface area, tunable porosity, and ease of functionalization. Such materials have been successfully employed in various applications, including separation and adsorption.^[^ ^40^, ^41^ ^]^ One such promising electrospun nanofibrous material is polyacrylonitrile (PAN), which has demonstrated excellent adsorption properties for various contaminants. PAN nanofibers possess a high surface area and can be easily modified to enhance their adsorption capabilities. Several studies have reported PAN nanofibers’ efficacy in removing organic dyes and pollutants from water, highlighting their potential for environmental remediation.^[^ ^42^, ^43^ ^]^

Herein, we present the development of a novel metal‐free adsorbent material by modifying PAN with semi‐crystalline nanocellulose (NC), creating a lightweight nanoweb through electrospinning. To address environmental and sustainability concerns, NC was derived from tissue paper waste as starting precursor. The addition of NC to the PAN matrix strongly improved the adsorption properties of the nanoweb, rendering it a highly efficient adsorbent for CV removal. Comprehensive characterization of the waste‐valorized nanowebs was carried out by scanning electron microscope (SEM), high‐resolution TEM (HRTEM), X‐Ray diffraction (XRD), thermogravimetry (TG), and total reflectance‐Fourier transform infrared (ATR‐FTIR). All materials can be recycled and the specific interactions between CV and the nanowebs were elucidated by Raman spectroscopy.

## 2 Results and Discussion

### 2.1 Synthesis and Characterization of Electrospun Nanowebs

**Figure 1:**
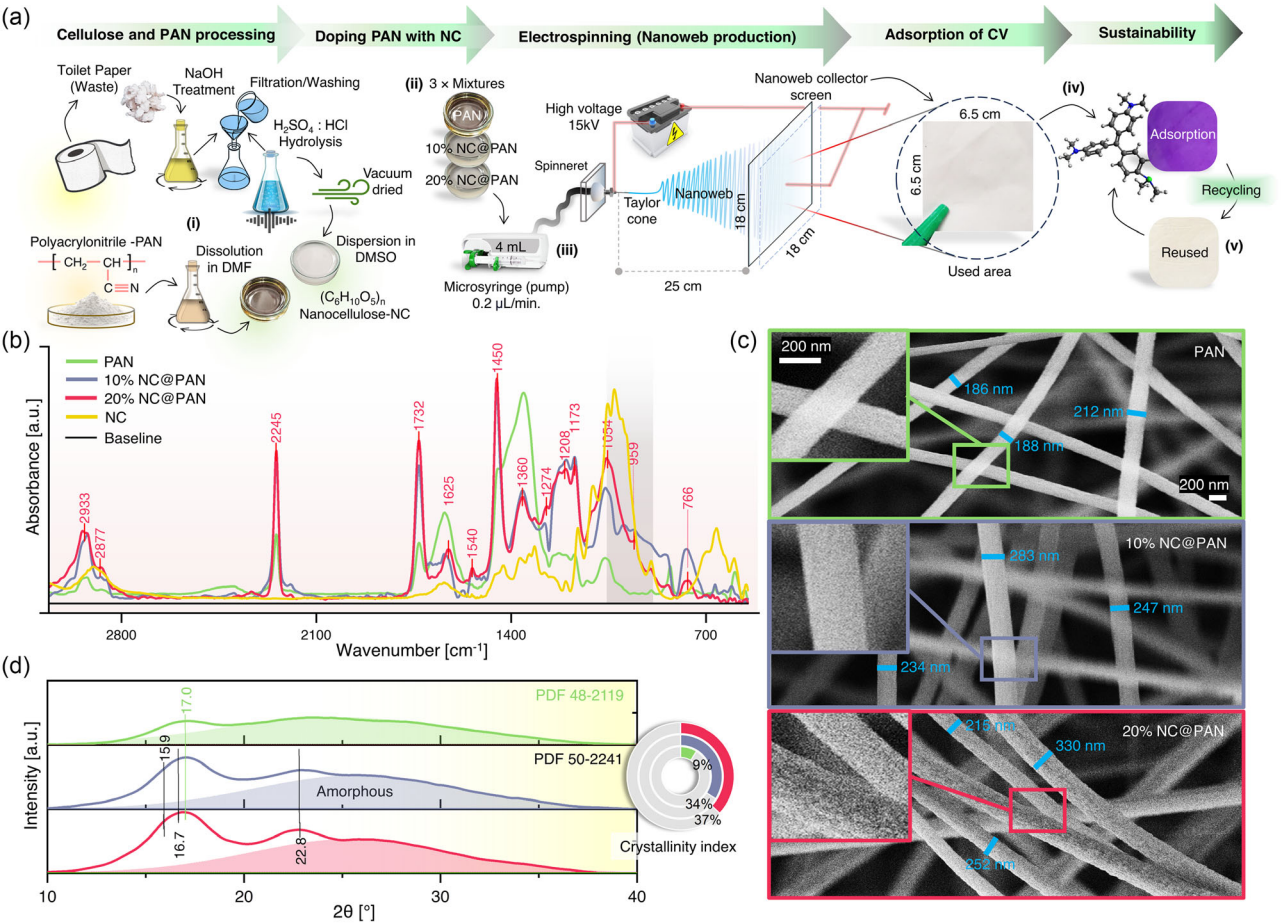
Synthesis and Characterization of Electrospun Nanowebs. a) Synthesis procedure showing i) processing of sanitary paper waste through alkaline treatment and acid hydrolysis to extract semicrystalline nanocellulose (NC), dispersion of NC into DMSO, and dissolution of polyacrylonitrile (PAN) in DMF, ii) preparation of three mixtures, that is, PAN, 10%NC@PAN, and 20%NC@PAN, iii) electrospinning of prepared mixtures to obtained nanowebs, vi) nanoweb as adsorbent for crystal violet from water, and v) sustainable process through recycling of nanoweb for reuse (see Experimental Section for details). b) ATR‐FTIR analysis of PAN, 10%NC@PAN, 20%NC@PAN, and NC. c) SEM images of as prepared PAN, 10%NC@PAN, and 20%NC@PAN (with magnified insets). d) X‐Ray diffraction pattern of PAN, 10%NC@PAN, and 20%NC@PAN, with a 2D pie chart showing the calculated crystallinity index (%).

The concept of using low‐cost waste to produce value‐added functionalized materials, in particular electrospun nanowebs, has not yet been fully recognized nor explored. Thus, we fabricated a series of sustainable electrospun nanowebs, mainly via modification of PAN with NC derived from tissue paper (organic) waste, as shown in **Figure** **1**a. After NaOH treatment, lignin‐containing impurities were removed from the biomass, followed by acid hydrolysis to extract semi‐crystalline (NC).^[^ ^44^, ^45^, ^46^ ^]^ The so‐obtained NC was mixed with dimethylsulfoxide (DMSO) to form a homogenous suspension, while PAN was dissolved in dimethylformamide (DMF). Later, three mixtures were prepared (pure PAN and modified with 10% and 20% of NC) and electrospun to obtain three different nanowebs (see Experimental Section for details).

To evaluate the chemical nature of the prepared nanowebs (PAN, 10%NC@PAN, and 20%NC@PAN), ATR‐FTIR spectroscopy was performed, as shown in Figure 1b. The bands at 2933 and 2877 cm^−1^ correspond to C—H in CH_2_ stretching (for assignments refer to the molecular structures illustrated in Figure S1, Supporting Information). The band at 2245 is related to the C≡N stretching of the acrylonitrile unit in the polymer chain. Absorption at 1732 cm^−1^ may be attributed to C=O in carbonyl group (of acetate group that could be generated by the reaction between PAN and water included in NC of nanofibers) and C=C vibrations.^[^ ^47^ ^]^ The nitriles react with adjacent groups to form cyclic carbon structures. These conjugated groups are characterized by an increase in the C=C and C=N bands at 1625 and 1540 cm^−1^, respectively.^[^ ^48^ ^]^ The band located at 1450 cm^−1^ characterizes C—H tensile vibrations in CH_2_ and that at 1360 cm^−1^ C—H stretching characteristics of aliphatic CH groups along the PAN backbone. The band at 1208 cm^−1^ is assigned to C—O vibrations in the acetate group, while that at 1054 cm^−1^ to C—O groups, connected with the carbon atoms of the 1, 2, 3‐propanetriol (by‐product of a reaction with water). The characteristic peaks in NC (894–1050 cm^−1^) may be assigned to C—O—C stretching at β‐(1 → 4)‐glycosidic linkages.^[^ ^49^, ^50^ ^]^ The existence of these bands in 10% and 20%NC@PAN clearly indicate that PAN had been successfully modified with NC.

SEM analysis was utilized to examine the structural properties and uniformity of the nanofibers forming the nanoweb. Figure 1c shows that the PAN sample consists of nanofibers with strand diameter in the range of 180–215 nm. It is apparent that the addition of NC to PAN induced two changes to the nanofibers, 1) the strand diameter of 10%NC@PAN was 245–285 nm and that of 20%NC@PAN was 215‐300 nm (i.e., both larger), 2) a higher surface roughness, likely due to presence of semicrystalline NC, also enhancing the contrast. All specimens show layered nanofibers, creating porosity due to nanofiber overlap, but without signs of agglomeration. Figure S2, Supporting Information, provides additional validation of the homogeneity of nanofibers in each specimen. Transmission electron microscopy (TEM) (Figure S3, Supporting Information) of PAN nanofibers showed that the surface has some structure/roughness but was relatively smooth when compared to other NC@PANs. HRTEM of PAN shows an amorphous homogeneous layer with slight contrast at the edges, which is related to the focused electron beam. While HRTEM of 10% NC@PAN and 20% NC@PAN both showed a pronounced contrast variation with respect to the coating layer, demonstrating that a darker PAN core is encapsulated by a certain thickness (lighter contrast) of NC. The thickness of the NC layer may vary due to the slight roughness of PAN. The surface roughness was also more noticeable in 20% NC@PAN due to the NC coating, in agreement with the SEM analysis.

Reports in literature suggest that the appearance of sharp ATR‐FTIR absorption bands (894 to 1050 cm^−1^) in NC, 10% and 20%NC@PAN could possibly be due to the crystallinity of NC.^[^ ^51^ ^]^ Indeed, XRD analysis confirmed the crystalline parameters of the prepared nanowebs, as displayed in Figure 1d. PAN showed a broad characteristic diffraction peak at 2*θ* = 17° (PDF 48‐2119). However, the major part is dominated by amorphous material (crystallinity index = 9%). 10% and 20%NC@PAN do not exhibit unified index before 2θ = 20°, probably due to the obscuring PAN signal in this region. Nevertheless, this broad peak may be assigned to 2*θ* = 15.9° (110) + 16.7 (110). The further detection of prominent diffraction at 2*θ* = 22.8° (200) suggests that the semicrystalline NC maybe of type‐I.^[^ ^52^ ^]^ Interestingly, the addition of NC to PAN improved the overall crystallinity index to 34% and 37% for 10%NC@PAN and 20%NC@PAN, respectively.

### 2.2 Adsorption of Crystal Violet

**Figure 2:**
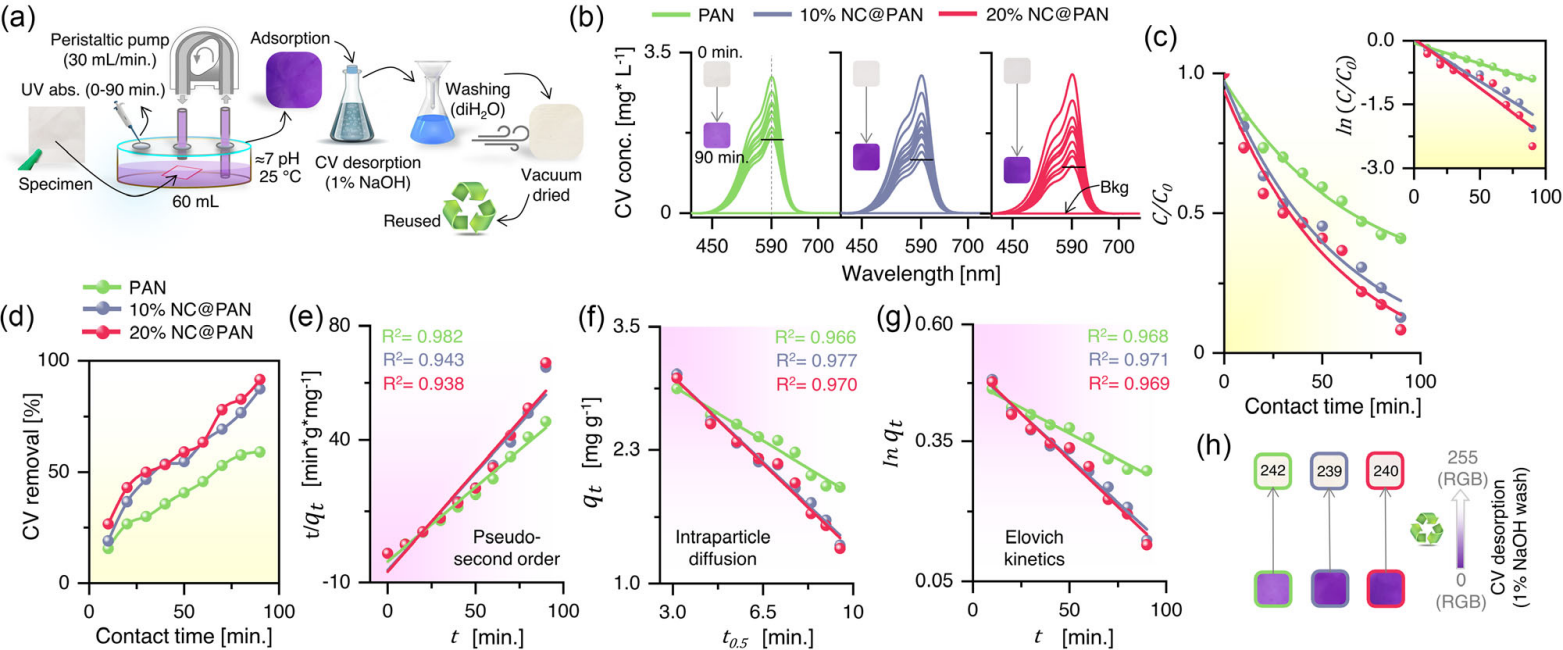
CV Adsorption Kinetics of Electrospun Nanowebs. a) Experimental setup for CV adsorption at room temperature using 6.5 cm^2^ of each nanoweb, immersed in 60 mL of CV‐aqueous solution (pH = 7, 3 mg L^−1^) for 90 min under continuous circulation b) Change in CV concentration due to adsorption at abs_max_ = 590 nm monitored up to 90 min by a UV spectrophotometer. A color change from white to violet is also obvious due to CV adsorption over the nanowebs. c) Change in CV concentration (*C/C* ^0^) over time (inset showing *In*(*C/C* ^0^)). d) CV removal, percentage over time. e) Pseudo second order kinetic model with linear correlation (*R* ^2^) values for each adsorbent. f) Intra‐particle diffusion kinetics model with linear correlation (*R* ^2^) values for each adsorbent g) Elovich kinetics model with linear correlation (*R* ^2^) values for each adsorbent h) CV desorption through 1% NaOH treatment, also showing RGB color changes (decolorization of the nanowebs due to CV removal).

The adsorption of CV was studied at room temperature, using 6.5 cm^2^ of each nanoweb immersed in 60 mL of CV‐aqueous solution (pH: 7, 3 mg L^−1^) for 90 min under continuous circulation, as shown in **Figure** **2**a. The change in CV concentration was monitored every 10 min by a UV spectrophotometer. The effect of contact time is decisive, because it directly influences the rate at which the adsorbate molecules are transferred from the solution onto the surface of the adsorbent nanowebs.^[^ ^53^ ^]^ During the initial stages of the adsorption process with the adsorbent surface becoming available for adsorption, there is typically a rapid decrease in the amount of dissolved CV molecules.^[^ ^54^ ^]^ This is obvious in Figure 2b–d, showing more CV adsorption at the start, which continues to increase over time, at a different rate, for all the nanowebs. As the contact time progresses, the adsorption rate gradually slows down, eventually reaching a saturation point where the amount of adsorbate being absorbed remains constant. This point indicates that the adsorbent surface has reached its maximum adsorption capacity, and no further adsorption occurs.^[^ ^55^ ^]^ Thanks to the excellent adsorption properties of NC,^[^ ^56^, ^57^ ^]^ addition of NC to PAN significantly improved the overall CV removal capacity. As expected, PAN showed with 59% the lowest CV removal in 90 min, while 10%NC@PAN showed 87%, and 20%NC@PAN showed the highest CV removal capacity of 91%.

The kinetic order and associated rate constants of CV adsorption were determined to better understand the adsorption process. Pseudo first order (PFO) (Equation (1))^[^ ^58^ ^]^ and pseudo second order (PSO) (Equation (2))^[^ ^59^ ^]^ models were applied to decide whether the rate of adsorption was directly related to the number of free adsorption sites or CV adsorption occurred due to of electrostatic (chemical) interactions between CV and the adsorbent.

PFO is expressed as

(1) $$\frac{dqt}{dt}=kf\left(q_{e}q_{t}\right)$$

where *q*_*t*_ (mg g^−1^) is the amount of adsorbate adsorbed at time *t*, *q*_*e*_ (mg g^−1^) is the amount of adsorbate at equilibrium time, and *k*_f_ is the pseudo first‐order rate constant. The linear form of PFO is

(2) $$\text{In}(q_{e}-q_{t})\;=\text{ In }q_{e}-k_{f}t$$

A plot In(*q*_*e*_ *− q*_*t*_) versus *t* (min) yields *q*_*e*_ and *k*_*f*_.

The PSO order is expressed as

(3) $$\frac{dqt}{dt}=k_{s}{\left(q_{e}-q_{t}\right)}^{2}$$

where *q*_*t*_ (mg g^−1^) is the amount of adsorbate adsorbed at time *t*, *q*_*e*_ (mg g^−1^) is the amount of adsorbate at equilibrium, and *k*_s_ is the pseudo second‐order rate constant. The linear form of the equation is

(4) $$\frac{t}{q^{t}}=\frac{1}{k_{s}q_{e}^{2}}+\frac{t}{q_{e}}$$

A plot $\frac{t}{q^{t}}$ (min g mg^−1^) versus *t* (min) yields *q*_*e*_ and *k*_s_.

The linear correlation (*R* ^2^) obtained from the PSO, as shown in Figure 2e, is 0.982, 0.943, and 0.938 for PAN, 10%NC@PAN, and 20%NC@PAN, respectively. *R* ^2^ obtained from the PFO only shows lower values (see Figure S4, Supporting Information), suggesting that time dependence of CV adsorption is much better described with PSO. Moreover, the higher *k*_s_ value (Table S1, Supporting Information) obtained for PAN (*k*_s_ = 1.4205) as compared to 10%NC@PAN (*k*_s_ = 0.3771) and 20%NC@PAN (*k*_s_ = 0.3264) further suggests that the adsorption on PAN occurred more rapidly, while addition of NC to PAN led to slower but continuous CV adsorption.

The multilayer alignment of nanofibers in PAN and NC‐modified PAN (as evident from Figure 1c) indicates that CV adsorption may also occur “in depth”, so that adsorbate molecules can possibly diffuse in between nanofibers multilayers, which can be evaluated through an intraparticle (Weber and Morris) diffusion model (IDM),^[^ ^60^, ^61^ ^]^ expressed as

(5) $$q_{t}=\;K_{id}t_{0.5}+\;I$$

where *q*_*t*_ (mg g^−1^) is the amount of adsorbate adsorbed at time *t*, *I* is the thickness of layer, and *K*_*id*_ (mg g^−1^ min^−1/2^) is the interparticle diffusion constant. A plot *q*_*t*_ (mg g^−1^) versus *t* ^0.5^ (min) was obtained to determine the *R* ^2^.

The slightly higher *R* ^2^ values (Figure 2f) of 10%NC@PAN (0.977) and 20%NC@PAN (0.970) as compared to PAN (0.966) confirm that CV adsorption is simultaneously occurring electrostatically over the surface, as well as in between the multilayered nanofibers.

Considering that CV adsorption in nanowebs follows PSO and IDM kinetic models, it seemed necessary to evaluate the applicability of an Elovich kinetic model (EKM),^[^ ^62^, ^63^ ^]^ which states that the adsorption process is controlled by chemisorption through surface and an initial rapid adsorption phase, followed by a gradual decrease in the rate as the surface becomes saturated. The equation for the EKM is

(4) $$q_{t}=(\frac{1}{\beta })\text{ ln}\left(\alpha \beta \right)+(\frac{1}{\beta })\text{In}t$$

where *q*_*t*_ is the amount of adsorbate adsorbed at time *t*, *α* is the initial adsorption rate constant (g g^−1^ min^−1^), and *β* is the desorption constant (g mg^−1^), which is related to the extent of surface coverage. Equation (4) was linearized and *lnq*_*t*_ plotted against *t*. *β* = 1/*e* ^^^(intercept) and *α =* slope*/β*, were *β* was determined from the intercept of the linear regression line and *α* from the slope of linear regression line.

The *R* ^2^ values (Figure 2g and Table S1, Supporting Information) for PAN, 10%NC@PAN and 20%NC@PAN are 0.968, 0.971, and 0.969, indicating CV chemisorption and once more supporting the PSO and IDM kinetic models. Desorption constants (*β*) for PAN, 10%NC@PAN and 20%NC@PAN were calculated as 0.6210, 0.6036, and 0.6011, respectively. The lower *β* values (≈0.60 g mg^−1^) indicate weaker bonding between the adsorbate and the adsorbent surface, leading to lower surface coverage and faster desorption. Figure 2h shows that the desorption of CV was successfully achieved by 1% NaOH treatment, enabling reusability of nanowebs in a following adsorption cycle.

Based on these results, an adsorption mechanism can be suggested. The adsorption of CV on NC modified PAN is collectively influenced by 1) multilayer adsorption among nanofibers, which describes that in the initial phase of adsorption CV molecules gradually diffuse into the inter‐space of nanofibers, overcoming steric hindrance. As a result, the rate (slope) at the end (60–90 min) is slower than at the start. The final equilibrium phase corresponds to multilayer diffusion, which exhibits the slowest adsorption rate. Hence, an equilibrium can be achieved either when all adsorption sites are fully occupied or when steric hindrance is encountered due to previously adsorbed CV molecules,^[^ ^61^, ^64^ ^]^ 2) chemisorption, involving van der Waals forces, electrostatic interactions, π–π interactions, and hydrogen‐bonding forces, with electrostatic interactions playing the primary role,^[^ ^54^ ^]^ and 3) presence of homogenous active sites, actively involved in the adsorption process.^[^ ^65^, ^66^ ^]^ Such multimodal adsorption kinetics has been well described in literature.^[^ ^65^ ^]^ However, considering the complexity of CV adsorption on nanowebs, this requires further experimental studies, motivating the confocal micro‐Raman spectroscopy below.

### 2.3 Mechanistic Study of Crystal Violet Adsorption

**Figure 3:**
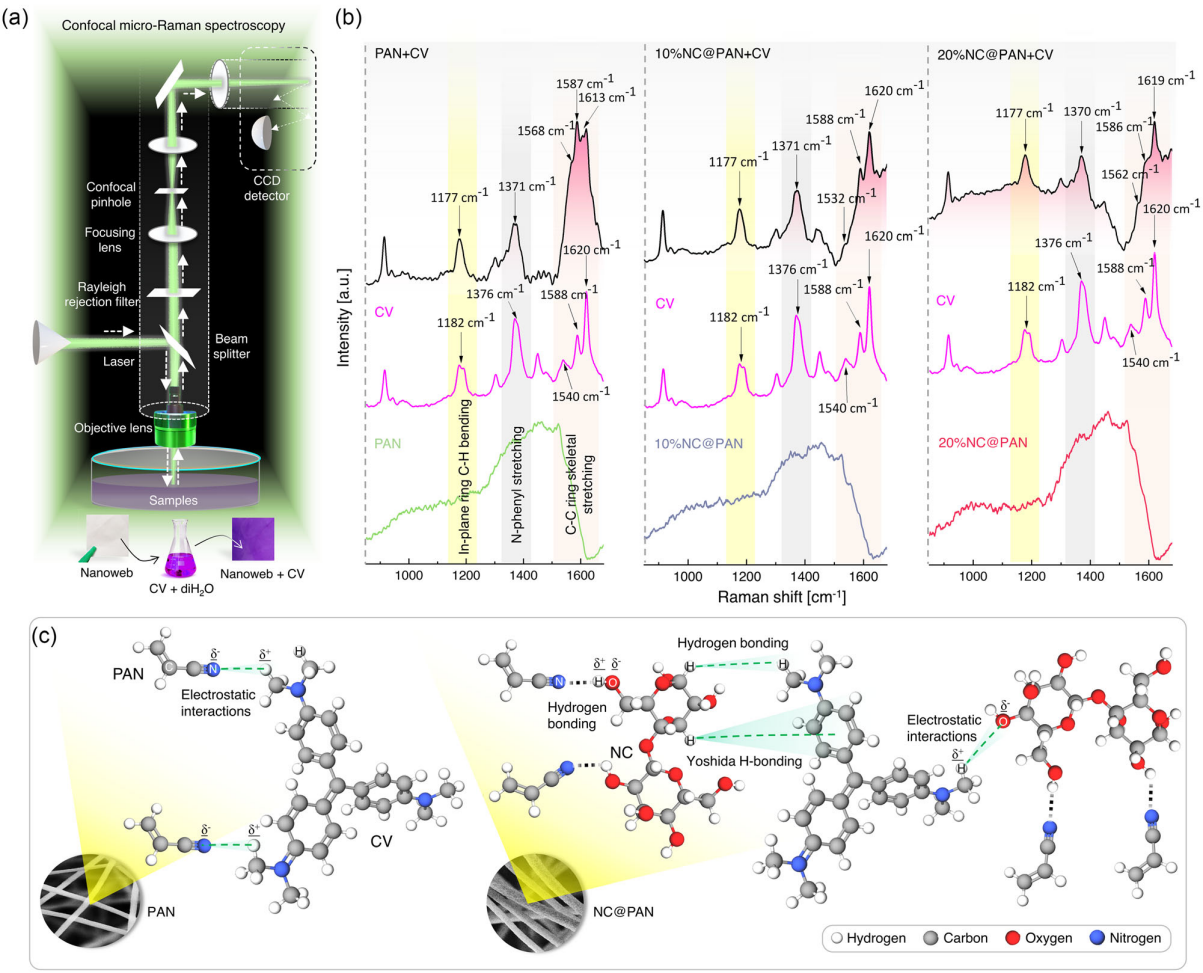
Mechanistic Study of CV Adsorption. a) Experimental design of confocal micro‐Raman spectroscopy of CV adsorption over nanowebs. b) Micro‐Raman spectra of CV/PAN, CV/10% NC@PAN, and CV/20% NC@PAN. Peak smoothing was performed using the adjacent averaging method with a window size of 6 and a polynomial order of 2. Cumulative Gauss fits (gray line) and corresponding deconvoluted peaks are selected based on the best *R* ^2^. PAN with dye (*R* ^2^: 0.97); 10% NC@PAN with dye (*R* ^2^: 0.97); and 20% NC@PAN (*R* ^2^: 0.98). c) A model showing CV adsorption mechanism by PAN and NC@PAN.

Confocal micro‐Raman spectroscopy was applied to compare the extent of CV adsorption by PAN, 10% NC@PAN, and 20% NC@PAN (**Figure** **3**a). In order to increase the accuracy of the analysis, for each sample the average of five random zones was taken. The Raman survey spectra obtained for all nanowebs before and after dye adsorption are shown in Figure S5, Supporting Information, and are discussed in more detail below.

As basis for post‐adsorption interaction, (pure) CV was also examined. Raman signals of CV were detected at 918 cm^−1^ (ring skeletal vibrations), 1182 cm^−1^ (in‐plane ring CH bending vibrations), and 1376 cm^−1^ (N‐phenyl vibrations). Ring C–C stretching vibrations were assigned to peaks at 1540, 1588, and 1620 cm^−1^.^[^ ^67^, ^68^ ^]^

First, PAN was studied before and after CV adsorption (Figure 3b). Before CV adsorption, peak deconvolution revealed peaks characteristic of C—C skeletal stretching (973 and 1186 cm^−1^), CH bending vibrations (1398 cm^−1^), CH_2_ bending (1516 cm^−1^), and CN (nitrile) stretching (2221 cm^−1^).^[^ ^69^ ^]^ After CV adsorption, the ring CC stretching intensities were higher (Figure 3b) than for pristine PAN, and they were detected at 1568, 1587, and 1613 cm^−1^. The stretching of the N‐phenyl group appeared at 1371 cm^−1^ and with a lower intensity than before. No distinct peak was detected for the nitrile group, but peak deconvolution led to an estimated signal at 2270 cm^−1^.

Next, PAN samples with 10% and 20% NC were subjected to Raman analysis before and after CV adsorption and compared with corresponding measurements of pristine PAN (Figure 3b). In general, Raman signals of NC can be observed in three regions: 3350–2700 cm^−1^, 1750–800 cm^−1^, and 610–150 cm^−1^.^[^ ^70^ ^]^ However, due to the low concentration of NC in the polymer samples and the overlap of peaks with those of PAN, peaks characterizing NC could not be identified. For example, the C—O—C stretching vibration of the glyosidic linkage of NC detected by FTIR (Figure 2b), including both symmetric and asymmetric vibrations (≈900–1200 cm^−1^),^[^ ^71^ ^]^ overlap with CC skeletal stretching of PAN. Therefore, NC‐modified PAN showed peaks similar to pure PAN, only with a slight shift.

Before adsorption, the 10% NC@PAN (Figure 3b) showed CC skeletal stretching vibrations at 976 and 1187 cm^−1^, CH bending at 1360 cm^−1^, and CH_2_ bending at 1497 cm^−1^. Nitrile stretching appeared at 2223 cm^−1^ with higher intensity after coating with 10% NC. After CV adsorption, a very intense and broad signal was observed at 2180–2369 cm^−1^. This may be due to the enhancement of CV adsorption on the NC‐modified PAN. Similar to PAN with CV, peak deconvolution estimated the nitrile signal at 2223 cm^−1^.

Before adsorption, 20% NC@PAN (Figure 3b) showed the CC skeletal stretching vibration at 1002 and 1158 cm^−1^, the CH bending vibrations at 1393 cm^−1^, and CH_2_ bending vibrations at 1532 cm^−1^. The intensity of the nitrile stretching (2218 cm^−1^) for 20% NC@PAN was almost as high as for 10% NC@PAN and higher than for PAN. In addition, prior to adsorption, all polymers exhibited a small peak (≈1700–1712 cm^−1^)^[^ ^72^ ^]^ associated with C=O in acetate (Figure S6, Supporting Information) analogous to FTIR, likely due to the reaction of PAN with water.

After adsorption, a red shift was observed for the in‐plane ring C—H bending (Figure 3b), associated with dye components (≈1177 cm^−1^). After adsorption, C—C ring skeletal stretching was also detected at 1532, 1588, 1620 cm^−1^, and 1562, 1586, 1619 cm^−1^, correspondingly, for both 10% NC@PAN and 20% NC@PAN (Figure 3b).

In addition, the N‐phenyl stretching of PAN and CV/20% NC@PAN after adsorption (Figure 3b) was red‐shifted from 1376 to 1371 cm^−1^. This may be caused by weakening of the N‐phenyl bond.^[^ ^73^ ^]^ However, the red shift was slightly stronger for CV/20% NC@PAN, with a corresponding signal appearing around 1370 cm^−1^. Presumably, there should be an interaction between CV via its nitrogen lone‐pair electrons with the hydroxyl group of NC, leading to a reduction in electron density of N via hydrogen bonding with the OH group^[^ ^74^ ^]^ in NC. Moreover, Yoshida H‐bonding^[^ ^75^ ^]^ can be expected to occur for CV/10% NC@PAN and CV/20% NC@PAN, between the aromatic rings of the CV and the hydroxyl group in the NC. Besides hydrogen bonding, electrostatic interactions are expected between the cationic part of CV and the negatively charged sites (e.g., hydroxyl groups) in NC. Thus, in NC@PANs, the electrostatic interactions can also be a factor in the increase of the CV adsorption rate.

Furthermore, after adsorption, a very intense and broad signal was again detected at 2201–2359 cm^−1^ (Figure S7, Supporting Information). The intensity of this signal was somewhat higher than that of CV/10%NC@PAN. Peak identification was not feasible, as the spectrum of CV at wavenumbers around 2400 cm^−1^ is not described in the literature. As a broad peak of nitrile was detected in all polymers after dye adsorption, the adsorption mechanism could also be due to a strong contribution of nitrile groups in polymers. Furthermore, the intensity of the nitrile peaks was about eight times higher than that of CV/PAN, suggesting that NC also contributed to the enhancement of interaction.

A model mechanism (Figure 3c) was therefore developed by analyzing the interactions of PAN with and without NC with CV using Raman spectroscopy to understand why NC could help to enhance the dye adsorption. Under normal experimental conditions, PAN without NC follow only physical interactions, in which the negatively charged nitrile groups on the polymer surface attract the positively charged dye molecules. This can be seen in the red shift of the N‐phenyl stretching and CH ring bending for CV after adsorption to PAN due to the decrease in electron density. The adsorption of CV onto the PAN surface is therefore by electrostatic attraction, and there were no significant chemical changes or reactions in either the PAN or the CV molecule that are involved in this physical interaction. Whereas, when NC is added to the composite, hydrogen bonding is expected to form between the lone pair electron of the nitrogen and the hydroxyl group in the NC. Then, as mentioned before, CV can be adsorbed by the NC on the PAN through physical (electrostatic) and chemical interactions.

### 2.4 Reusability and Stability of Electrospun Nanowebs

Industrial applications rely on stable and reusable adsorbents. Consequently, tests were conducted to assess the recycling performance of the nanowebs, and their stability was evaluated by SEM, TG, and XRD analysis. In this particular type of experiment, CV‐loaded adsorbents were introduced into a 1% NaOH solution.^[^ ^76^ ^]^ According to literature, NaOH facilitates the release of CV,^[^ ^77^ ^]^ possibly through weakening the electrostatic attractions between CV and the nanoweb and formation of a water‐soluble complex in the presence of OH ions. Still, the precise mechanism remains a topic of debate.^[^ ^78^ ^]^ The solution was vigorously stirred for 30 min, followed by drainage, thorough washing with diH_2_O, recovery of the nanowebs (Figure S8, Supporting Information), which were finally vacuum dried. The entire process was repeated twice.

**Figure 4:**
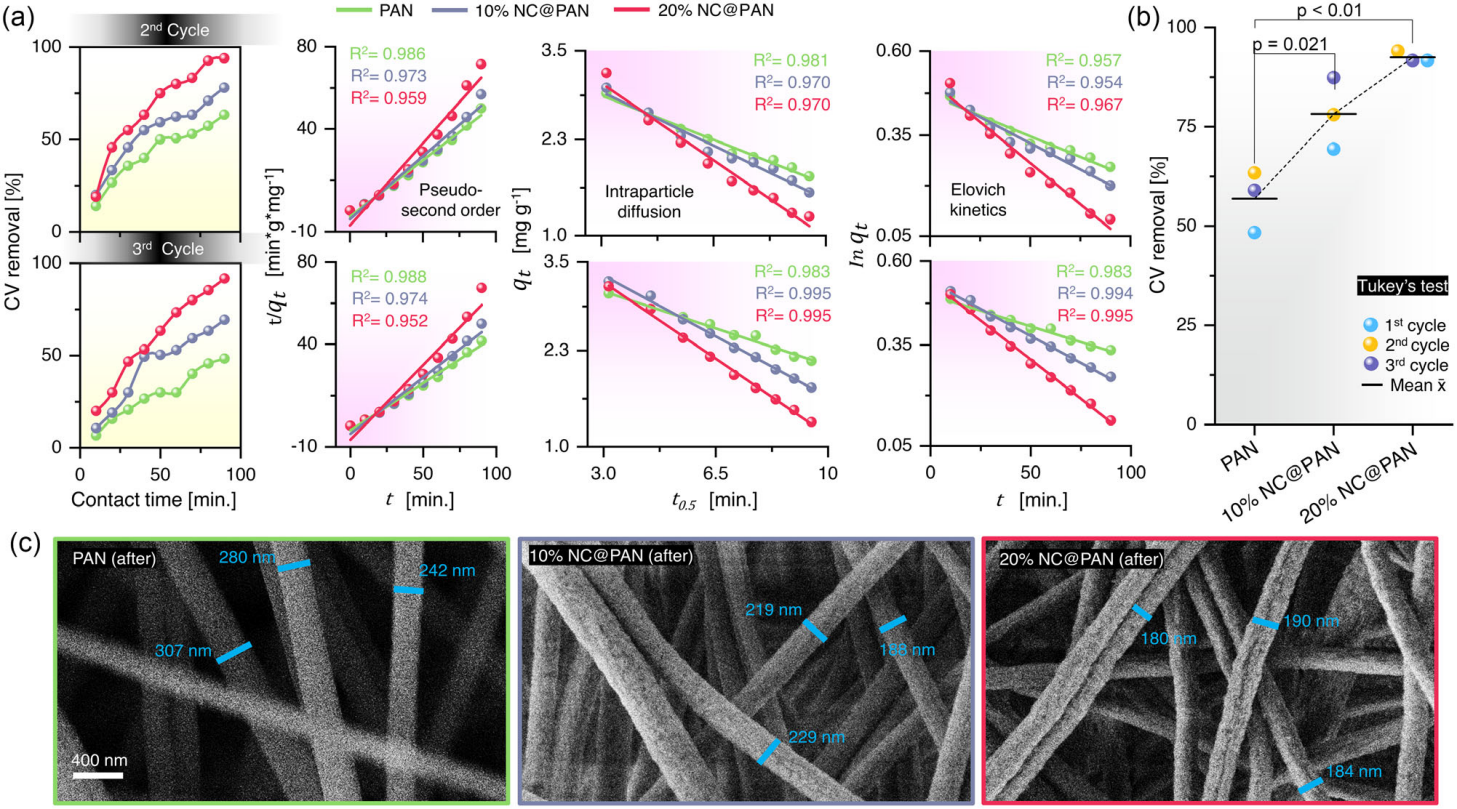
Reusability and Stability of Electrospun Nanowebs. a) CV removal percentage, linear correlation (*R* ^2^) values for pseudo second order, intra‐particle diffusion and Elovich kinetics model (see Figure S9 and S10, Supporting Information, for UV‐vis spectra and In(*C/C*_*0*_) of CV adsorption over time). b) Tukey's test on CV‐removal percentage data of nanowebs after three adsorption cycles. c) SEM analysis of PAN, 10% NC@PAN and 20% NC@PAN after three CV‐adsorption cycles.

Apparently, 20% NC@PAN showed (**Figure** **4**a) consistent performance with 91–94% CV removal over the repeated cycles, which confirmed a reversible adsorption process. In contrast, PAN had the lowest CV removal percentage, reaching 48% in the third cycle. Still, all nanowebs exhibited significant linear fit to PSO, IDM, and EKM adsorption kinetic models (Table S1, Supporting Information). Additionally, Tukey's test was performed^[^ ^79^ ^]^ (Figure 4b) to determine if PAN, 10% NC@PAN, and 20% NC@PAN were statistically significantly different from each other regarding CV adsorption over repeated cycles. Comparing 20% NC@PAN with PAN, *p* < 0.01 was obtained indicating that there is a substantial difference in CV adsorption performance between these two, whereas *p* = 0.021 when comparing 20% NC@PAN with 10% NC@PAN.

SEM analysis (Figure 4c) after three CV adsorption cycles confirmed the structural stability of all nanowebs. However, PAN exhibited an increase (≈36%) in nanofiber strand diameter, possibly due to H_2_O or NaOH treatment causing swelling. 10% NC@PAN and 20% NC@PAN did not show any pronounced morphology changes, further confirming the role of NC as stabilizer for better PAN performance.

**Figure 5:**
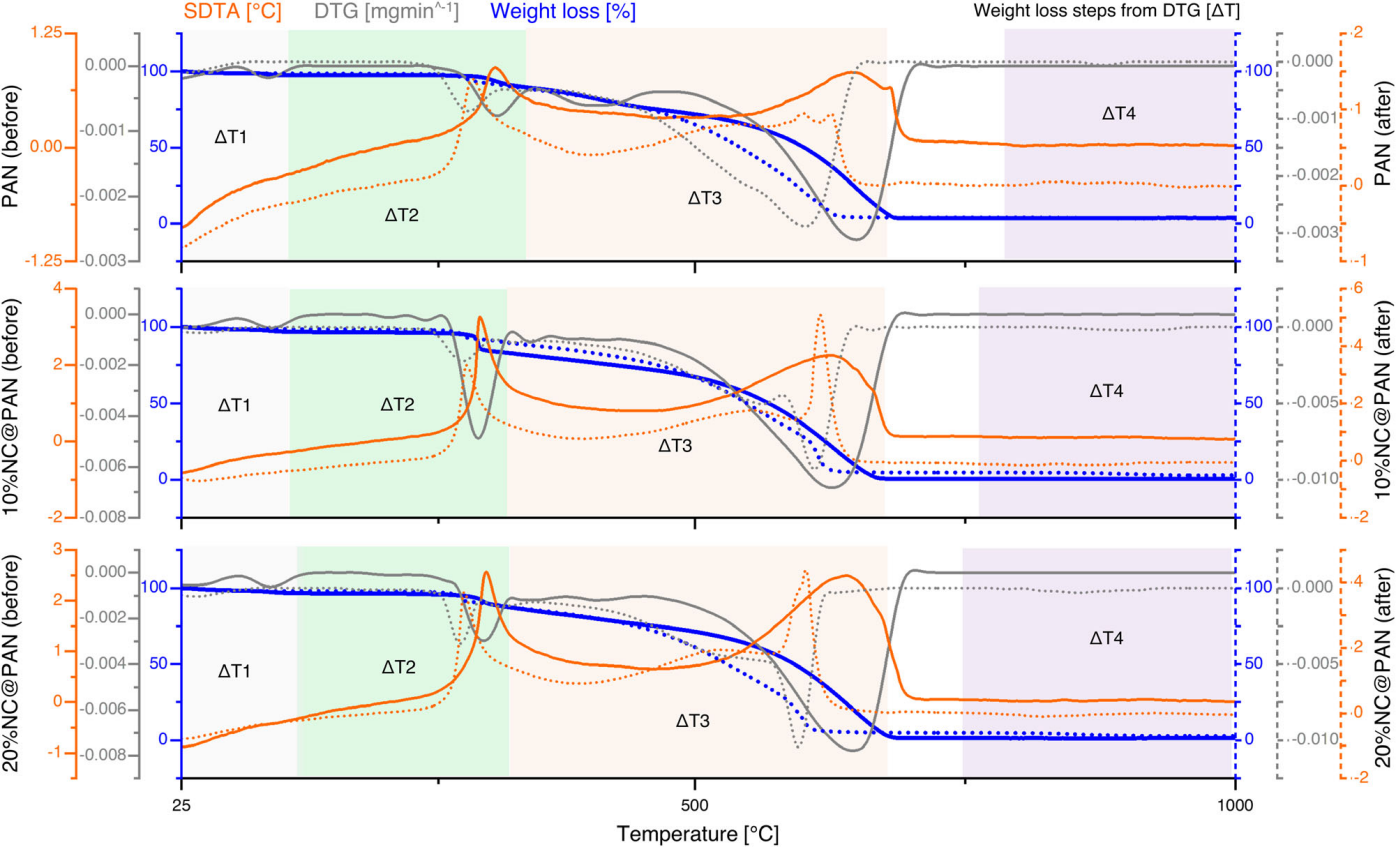
TG, DTG, SDTA curves, and weight loss steps from DTG (Δ*T*) of PAN nanowebs. 0%, 10%, and 20% NC before (colored lines) and after (colored, dotted lines) exposure to CV (more details in Figure S11, Supporting Information).

The structural and thermal stability of the nanowebs before and after CV adsorption were further analyzed through TG, as shown in **Figure** **5**. Briefly, the obtained TG, derivative thermogravimetry (DTG) and simultaneous differential thermal analysis (SDTA) curves (Figure 5) were analyzed in order to measure, for each specimen, the decomposition temperature Td, defined as the temperature of 5% mass loss, the main temperature intervals (ΔTn) with the corresponding weight loss and the corresponding temperatures of maximum decomposition (Tn), if detected, as reported in **Table** **1**.

**Table 1:** Thermal properties (main temperature intervals ΔTn, corresponding weight loss, temperatures of maximum decomposition (in brackets, if observed), under air of PAN nanowebs with 0%, 10%, 20% nanocellulose before and after exposure to CV

Specimen		Before				After				
		*T*_d_	ΔT1	ΔT2	ΔT3	Td	ΔT1	ΔT2	ΔT3	ΔT4
PAN	T [°C]	312	40–214 (105)	214–473 (314)	473–1000 (648)	292	37–199	199–357 (290)	357–788 (598)	788–1000
	−Δm [%]	5	1.9	23.3	70.9	5	1.0	11.1	83.5	0.6
10%NC@PAN	T [°C]	293	37–167 (107)	167–423 (302)	423–1000 (630)	287	36–229	229–356 (287)	356–761 (600)	761–1000 (904)
	−Δm [%]	5	2.9	21.3	74.6	5	2	10.8	82.8	1.8
20%NC@PAN	T [°C]	293	41–160 (107)	160–454 (305)	454–756 (647)	284	38–228	228–355 (285)	355–750 (601)	750–1000 (859)
	−Δm [%]	5	2.8	20.7	74.4	5	1.8	12.3	80.4	2.1

In nanowebs, under air, thermal decomposition occurs first with an endothermic peak; then oxidative decomposition appears with an exothermic peak. However, during the first weight decrease, it is likely that endothermic and exothermic peaks overlap, and thermal and oxidative decomposition occur back‐to‐back.^[^ ^80^ ^]^ In the case of PAN, TGA analysis showed three distinct main weight loss stages: approximately, the first stage is from room temperature to 200 °C, the second stage from 200 to 450 °C, and the last stage from 450 to 1000 °C.

In particular, in this case, the first endothermic stage from room temperature to about 200 °C, with maximum degradation rate around 100 °C, can be attributed to the evaporation of small molecules such as physically adsorbed water (dehydration) and removal of organic solvent residues trapped in the body of hollow nanofibers. The weight loss is very small, always lower than 3% of the total weight loss.

The second step began at *T* > 160 °C when the exothermal thermal decomposition of PAN nanoweb started. In particular, in this case, Td is 312 °C for PAN nanoweb, and it decreases by about 20 °C with NC, regardless of amount, meaning that the thermal stability of nanowebs is slightly affected by NC addition. Thermal decomposition of PAN accelerated up to 314 °C for pure PAN and up to 302 and 305 °C for 10%NC@PAN and 20%@PAN, respectively. During this step, the rate of weight loss became quite rapid, and it was mainly due to the PAN dehydrogenation accounting for about 23% of the total weight in pure PAN web and for 21% in PAN with NC.

In the last step, at *T* > 420 °C, on the one hand, fragmentation of polymer chains due to the bond breaking through the polymer chain of PAN occurred and, on the other hand, oxidation reactions led to the evolution of small volatile molecules such as NH_3_, NO_2_, HCN, CO, CO_2_, and H_2_O.^[^ ^81^ ^]^ The temperature of maximum decomposition for this stage was 648 °C for PAN and 630 °C and 647 °C for 10%NC@PAN and 20%@PAN, respectively. The thermal decomposition of PAN was mainly concentrated in this third stage with about 70% of weight loss. The residues were 3–4%.

After aging (reuse of nanowebs for up to three CV‐adsorption cycles), as expected, the first endothermic stage from room temperature to about 200 °C, attributed to the evaporation of small molecules, was reduced. In aged specimens also the thermal stability of PAN nanowebs slightly decreased since *T*_d_ lowered from 312 to 292 °C for PAN (Δ*T* = 20 °C) and from 293 to 287 °C and from 293 to 284 °C (Δ*T* = 6 °C) for 10%NC@PAN and 20%NC@PAN, respectively. Also, the peak of maximum decomposition of the second exothermic step shifted to lower temperatures: from 314 to 290 °C for PAN and from 302 to 287 °C and from 305 to 285 °C for 10%NC@PAN and 20%@PAN, respectively.

In the third step, the temperature of maximum decompositions of aged nanowebs was again lower than those of unaged ones, moving from 648 to 598 °C for PAN and from 630 and 647 °C to 600 and 601 °C for 10%NC@PAN and 20%NC@PAN, respectively. In the aged specimens, at about 850 °C, an additional very small weight loss of about 2% appeared probably due to the decomposition of Na_2_CO_3_ generated by the reaction of residual NaOH, with which specimens had contact two times, with atmospheric CO_2_.^[^ ^82^, ^83^, ^84^, ^85^ ^]^ The total weight loss remained around 97% for all specimens.

However, TG analysis (Figure 5) and XRD (Figure S12, Supporting Information) did not show any important changes, before and after CV adsorption cycles, indicating overall thermal and structural stability of all nanowebs. The overall CV adsorption performance of NC modified PAN was found to be competitive to the ones reported in literature (see Table S2, Supporting Information).

## 3 Conclusions

The current study demonstrates the exceptional adsorption performance of electrospun PAN nanowebs that can be further modified with NC derived from waste tissue paper, enabling efficient removal of CV from aqueous solutions. Notably, the nanoweb formulation containing 20% NC achieved stable and exceptional CV removal rates ranging from 91% to 94% over multiple cycles, even when dealing with trace amounts of CV at 3 mg L^−1^. This clearly demonstrates the outstanding efficiency of this adsorbent, which can effectively clean water from extremely low concentrations of contaminants, which is more realistic for industrial needs; other adsorbents often require higher initial adsorbate concentrations for optimal function.

Moreover, kinetic analysis revealed that the CV adsorption process predominantly follows a pseudo‐second‐order model, emphasizing the critical role of chemical interactions. These chemical interactions driving the adsorption mechanism were mainly attributed to the nitrile group of polymers in general, and between CV and NC, via its nitrogen lone‐pair electrons interacting with the hydroxyl group of NC. This interaction leads to a reduction in electron density of N through hydrogen bonding with the OH group of NC, further influencing the adsorption mechanism. Confocal micro‐Raman spectroscopy further illustrated the complexity of the adsorption process, implicating both surface adsorption and multilayer diffusion, with NC amplifying the interaction between CV and the nanowebs. The versatility of the observed adsorption mechanism, supported by statistical and numerical evidence, extends its relevance beyond CV removal, offering promising avenues for addressing diverse organic contaminants/dyes in water treatment. To advance our understanding of the CV adsorption process under different environmental conditions, future research should also explore adsorption isotherms, pH, and temperature dependency.

## 4 Experimental Section

Sterile ultrapure water (diH_2_O) by Milli‐Q, PAN, CV dye, NaOH, HCl, H_2_SO_4_, DMSO, and DMF by Merck, tissue paper as a potential organic waste (Università Politecnica delle Marche, Ancona, Italy), Electrospinning system with flat plate collector (SPINBOX, Italy), SEM coupled with energy‐dispersive X‐Ray spectroscopy (EDXS) (ZEISS SUPRA‐40 and X‐Flash Detector 410‐M by Bruker Nano GmbH), transmission electron microscope (FEI Tecnai F20 S‐TWIN), ATR‐FTIR spectrometer (Perkin–Elmer), SDTA‐TGA (Mettler‐851), hotplate magnetic stirrer (RH basic, IKA, Germany), water bath ultrasonication (FALC, Italy), heating oven (LA PORTA, Italy), centrifuge (EC‐CL31R Multispeed, Thermo Scientific, USA), XRD by X’Pert^3^Powder, PANalyticalTGA (Mettler‐851), UV/Vis spectrometer (Shimadzu UV‐1900i), and Confocal micro‐Raman spectroscopy (Horiba XploRA INV).

4.1

4.1.1

##### Synthesis Procedures

1) Extraction of semi‐crystalline NC was obtained following a previously reported method,^[^ ^86^, ^87^ ^]^ with modifications (Figure 1a). Briefly, tissue paper waste was finely chopped into small pieces (≈0.5 cm) using scissors. The chopped pieces were placed in 6% NaOH solution overnight. The obtained product was then filtered at room temperature using Whatman filter paper No.1. The residue was subsequently washed with diH_2_O to remove the NaOH residue and the whitish cellulose obtained from the process was dried in a desiccator. To obtain NC, acid hydrolysis was performed by treating the dried cellulose with 500 mL of 5% H_2_SO_4_ : HCl (1:1): the mixture was first stirred over a hot plate magnetic stirrer for 2 h at 80 °C and then ultrasonicated for 1 h at 70 °C. The obtained NC was filtered using Whatman filter paper No.1, washed three times with diH_2_O to remove acid residues, and dried in a desiccator. Next, 1.5 g of NC was added to 41 mL of DMSO and sonicated at 70 °C until NC was completely dispersed in DMSO (≈1 h). The obtained NC suspension was transferred to an airtight jar for further usage. 2) Next, 4.87 g PAN powder was dissolved in 50 mL DMF. The process was carried out while stirring over a hot plate magnetic stirrer at 90 °C and 360 rpm for 1 h. A semi‐transparent PAN suspension was obtained at the end and transferred to an airtight jar for further usage. 3) For electrospinning of nanowebs, three premixtures (specimens) were made a) as prepared PAN suspension (4 mL) b) 10% NC in PAN, stirred, sonicated for 15 min (4 mL) c) 20% NC in PAN, stirred, sonicated for 15 min (4 mL)

For more details on mixtures design, please refer to Note S1, Supporting Information.

Electrospinning was performed at a flow rate of 0.02 μL/min with a target volume of 4 mL and voltage difference of 15 kV. The distance between the spinneret and flat plate collector (18 cm^2^) was 24 cm. Humidity was 50% at ambient temperature. The obtained nanowebs were stored in a desiccator for further use.

##### Characterization of Prepared Nanowebs

Attenuated total reflectance–Fourier‐transform infrared spectroscopy (ATR‐FTIR) was employed to obtain vibrational spectra of as prepared nanowebs and NC. Absorption spectra, as a result of 32 scans (range 530–4000 cm^−1^), were collected on an IR grade ZnSe crystal, in reflectance mode. Background spectra were acquired on clean regions of the silica windows. Baseline (polynomial line fit) and smoothing were performed in all cases using OriginPro software. ATR‐FTIR was done to determine the NC structure and impact of NC on PAN modification.

XRD analysis enabled to analyze the crystallographic parameters of all specimens (nanowebs), using a Cu‐α radiation source (λ = 1.5406 Å), carried out at room temperature with an operating voltage of 40 kV and a current of 30 mA, covering an angular range of 5°–85° (2*θ*) of diffraction angles. Baseline corrections and smoothing were performed in all cases using OriginPro software.

Moreover, scanning electron microscopy (SEM) was employed to image the microstructural properties of all nanowebs before and after three CV adsorption cycles. Briefly, the dried specimens were immobilized over commercial Cu grids, further coated with Au for SEM observation. Micrographs were taken at 10–20 kV and at high resolution (magnification = 260–475 kX, aperture size = 30 μm).

PAN, 10% NC@PAN, and 20% NC@PAN samples slightly moistened with distilled water were placed on lacy carbon Cu grids and vacuum dried before examination by TEM and high‐resolution transmission electron microscopy (HRTEM), on a FEI Tecnai F20 S‐TWIN at 200 kV. Images were captured and processed using Gatan microscopy suite software (DigitalMicrograph).

TG, DTG, and SDTA of different PAN nanowebs were carried out both before and after CV treatment in order to investigate the effect of NC addition and aging on the thermal properties of specimens. Tests were carried out under air flux set at 60 mL min^−1^ in the temperature range 25–1000 °C and increase rate of 10 °C min^−1^. For each sample, 5–20 mg of the dried powder sample was taken in an alumina‐150 μL sample holder.

Confocal micro‐Raman spectroscopy was performed at room temperature^[^ ^88^ ^]^ using a Horiba XploRA INV. The Raman system was equipped with a light microscope (Nikon Eclipse TiU), an XY motorized stage and a thermo‐electrically cooled charge‐coupled device detector. A diode laser (532 nm) with 100% (pre‐adsorption) and 25% (post‐adsorption) intensity was used as the excitation source. The beam was focused onto a sample using a 5× objective. Raman spectra were recorded between 0 and 3000 cm^−1^ by using a holographic grating of 1800 grooves mm^−1^, an acquisition time of 1 s, accumulation of 12 measurements, a slit of 200 μm, and aperture of 300 μm. To calibrate and collect the spectra, HORIBA Scientific's LapSpec6 spectroscopy suite software was used. Signal processing and peak smoothing were performed by applying the adjacent averaging method with a window size of 6 and a polynomial order of 2. For the micro‐Raman analysis of polymers and liquids, μ‐dish holders (Ibidi) with a glassy bottom and a diameter of 35 mm were used. Water was drop casted onto the samples in a μ‐dish to prevent samples from being burned by the laser due to the high absorbency of the CV dye.

##### CV Adsorption Studies

The potential of nanowebs as promising adsorbent materials for removal of organic pollutants from water was tested by using 60 mL of CV aqueous solution (3 mg L^−1^). Specifically, three specimens were tested, PAN (57 mg), 10% NC@PAN (53 mg), and 20% NC@PAN (50.6 mg). The calculation of NC to PAN weight ratio is further detailed in Note S1, Supporting Information, and the experimental design is illustrated in Figure 2a. Briefly, each specimen was placed in 60 mL of CV solution (3 mg L^−1^), under continuous circulation (30 mL min^−1^) for 90 min at room temperature and pH 7. After every 10 min, the CV solution was collected and tested by UV‐visible absorption spectroscopy in the range 200–800 nm. To ensure a constant volume of CV solution, after each UV‐vis test, the CV solution was put back. Change in concentration of CV was recorded and plotted in OriginPro (v8.5) considering diH_2_O as blank (constant for all specimens) and CV solution (untreated) as control. All observations were made in triplicate, in the presence of blank and control, and averaged results are reported herein. Baseline correction, blank subtraction, and calibration of at least seven different concentrations of CV solution (Figure S10, Supporting Information) were performed for all UV‐Vis experiments (software “LabSolutions UV‐Vis v1.12” by SHIMADZU®). Furthermore, pseudo‐first order, pseudo‐second order, interparticle diffusion, and EKMs were applied on the obtained data, and *R* ^2^ (linear correlation) is reported in all cases.

For the reusability experiments, CV‐loaded adsorbents (nanowebs) were introduced into a 1% NaOH solution.^[^ ^76^ ^]^ The solution was vigorously stirred for 30 min, followed by drainage, thorough washing with diH_2_O, and the recovery of the nanowebs. The recovered nanowebs were then subjected to vacuum drying. Each reusability experiment was repeated two times. Finally, all CV adsorption data were compared, and Tukey's test was performed to evaluate possible differences between the adsorbents with respect to repeated CV adsorption.

## Conflict of Interest

The authors declare no conflict of interest.

## Author Contributions

Q.M.: Conceptualization, methodology, validation, software, formal analysis, investigation, data curation, writing—original draft, writing—review and editing. I.C.: Methodology, validation, formal analysis, investigation, writing—review and editing. N.L.: Methodology, validation, formal analysis, investigation, data curation, review and editing. S.S.: Methodology, validation, formal analysis, investigation, data curation, writing—review and editing. F.T.: Conceptualization, methodology, resources, writing—review and editing, supervision. G.R.: Conceptualization, methodology, resources, writing—review and editing, supervision.

## Supporting information

Supplementary Material

## Data Availability

The data that support the findings of this study are available from the corresponding author upon reasonable request.
