# Supplementary material for: Waste‐Valorized Nanowebs for Crystal Violet Removal from Water
Source: Small Sci. 2024 Feb 2;4(4):2300286. doi: 10.1002/smsc.202300286 (PMC11935146; doi:10.1002/smsc.202300286)
Supplement: Supplementary file 1 — Supplementary Material [file SMSC-4-2300286-s001.pdf]

Supplementary Information

Waste-Valorized Nanowebs for Crystal Violet Removal from Water

Qaisar Maqbool[1,2], Isabella Cavallini[1], Niusha Lasemi[2], Simona Sabbatini[1], Francesca Tittarelli[1], and Günther Rupprechter[2*]

[1]Department of Materials, Environmental Sciences and Urban Planning (SIMAU), Università Politecnica delle Marche, INSTM Research Unit, via Brecce Bianche 12, 60131 Ancona, Italy.
[2]Institute of Materials Chemistry, TU Wien, Getreidemarkt 9/BC, A-1060 Vienna, Austria.

*Correspondence to guenther.rupprechter@tuwien.ac.at

Number of pages: **S1-S19**

Number of supplementary Figures: **S1-S13**

Number of supplementary Tables: **S1-S2**

Number Supplementary Notes: **S1**

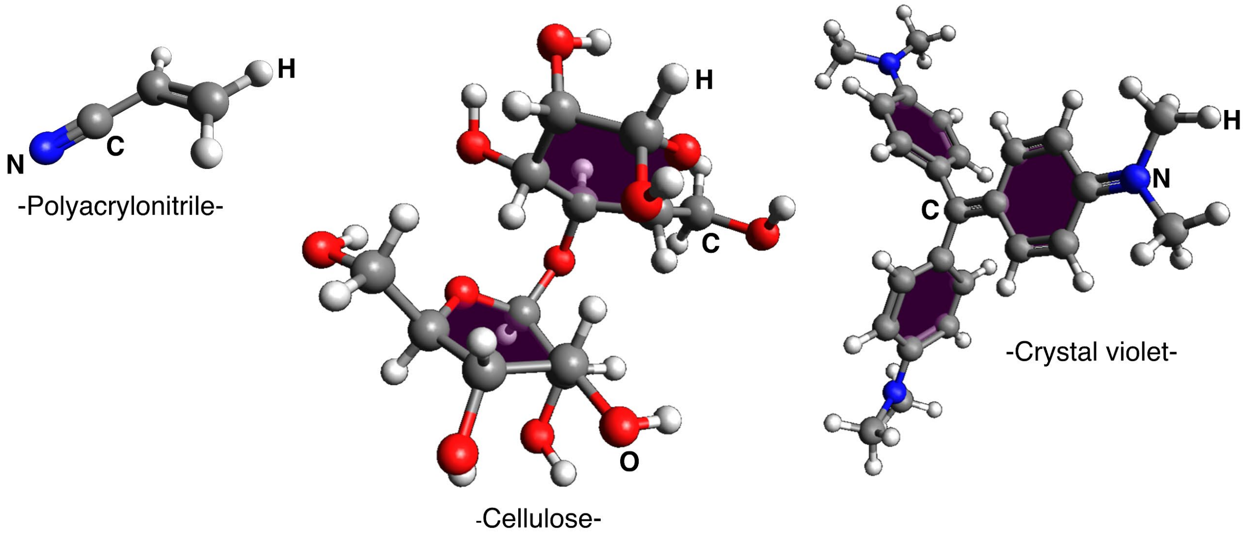

Figure S1. Molecular structures of PAN, NC and CV.

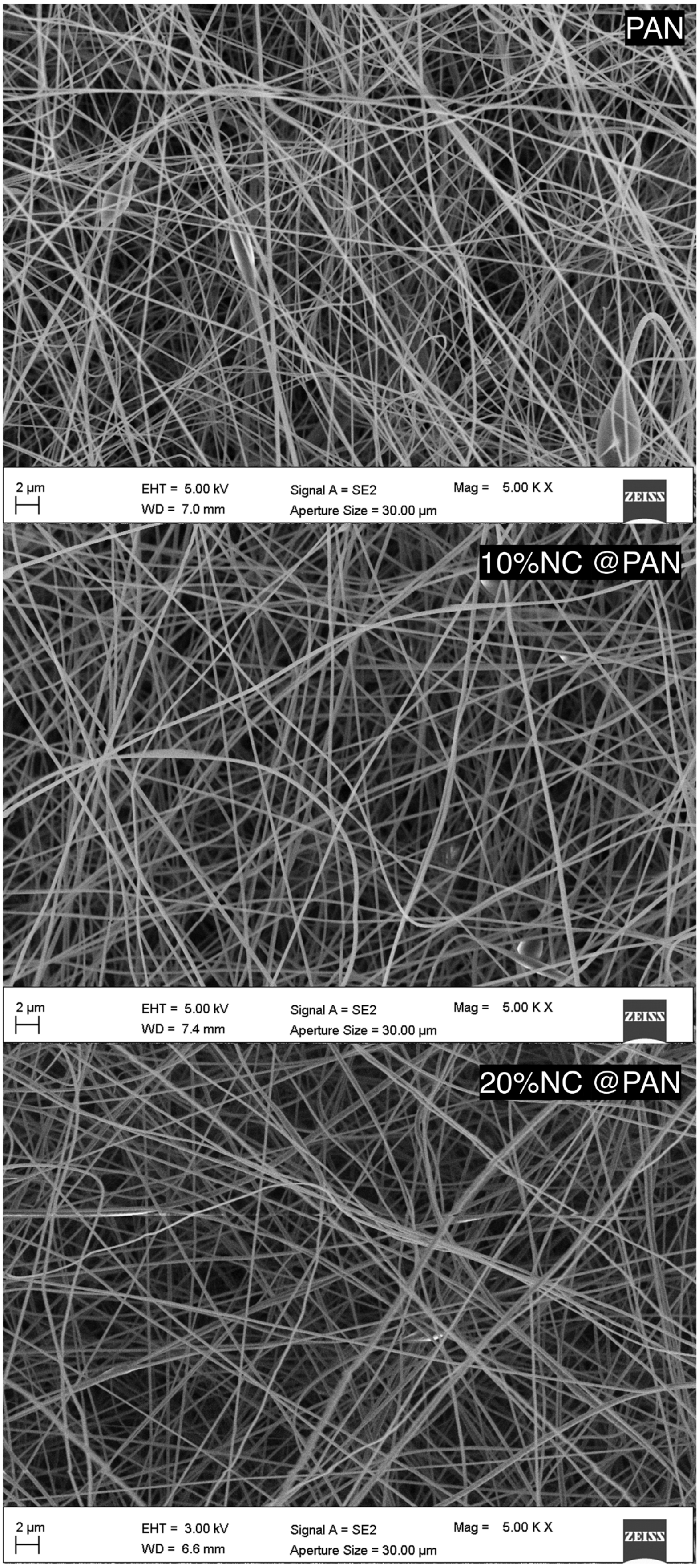

Figure S2. SEM of PAN, 10%NC@PAN, and 20%NC@PAN.

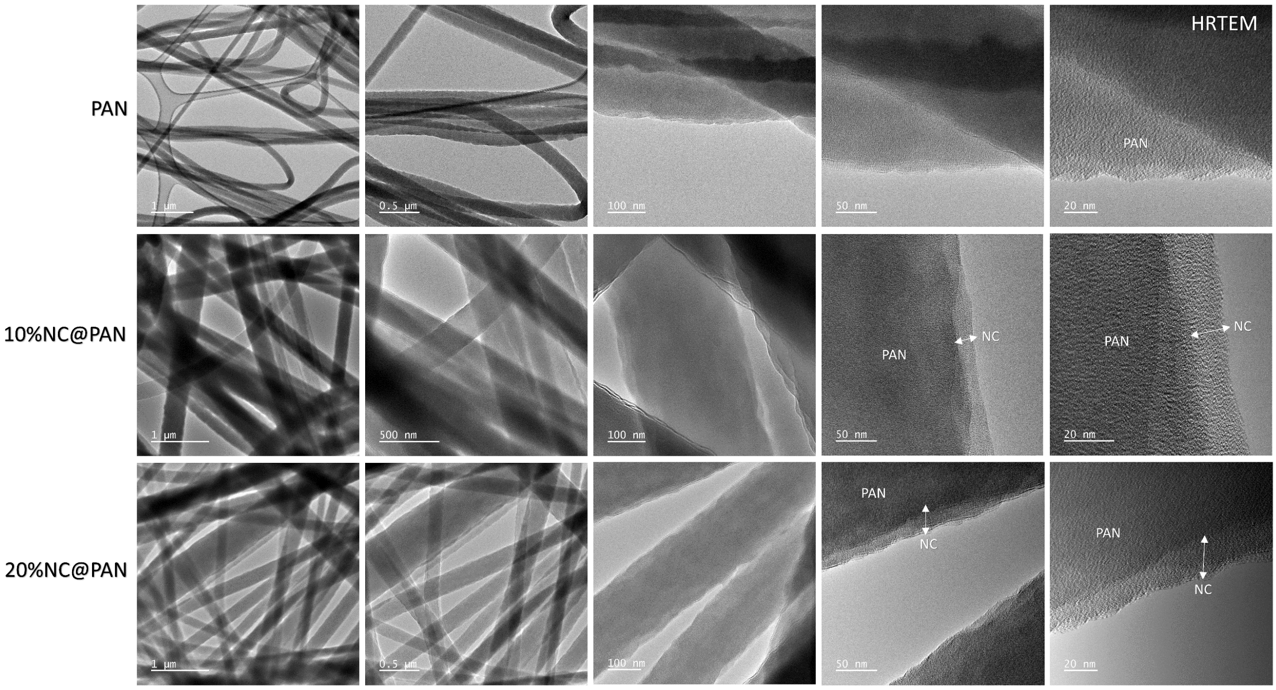

Figure S3. Bright field TEM and HRTEM of PAN, 10%NC@PAN, and 20%NC@PAN.

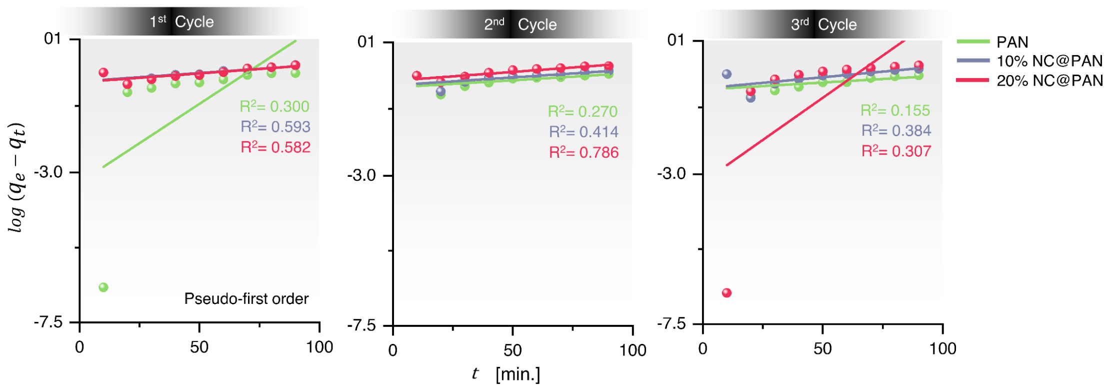

Figure S4. Pseudo first order kinetics model with linear correlation (R^2) values for each adsorbent.

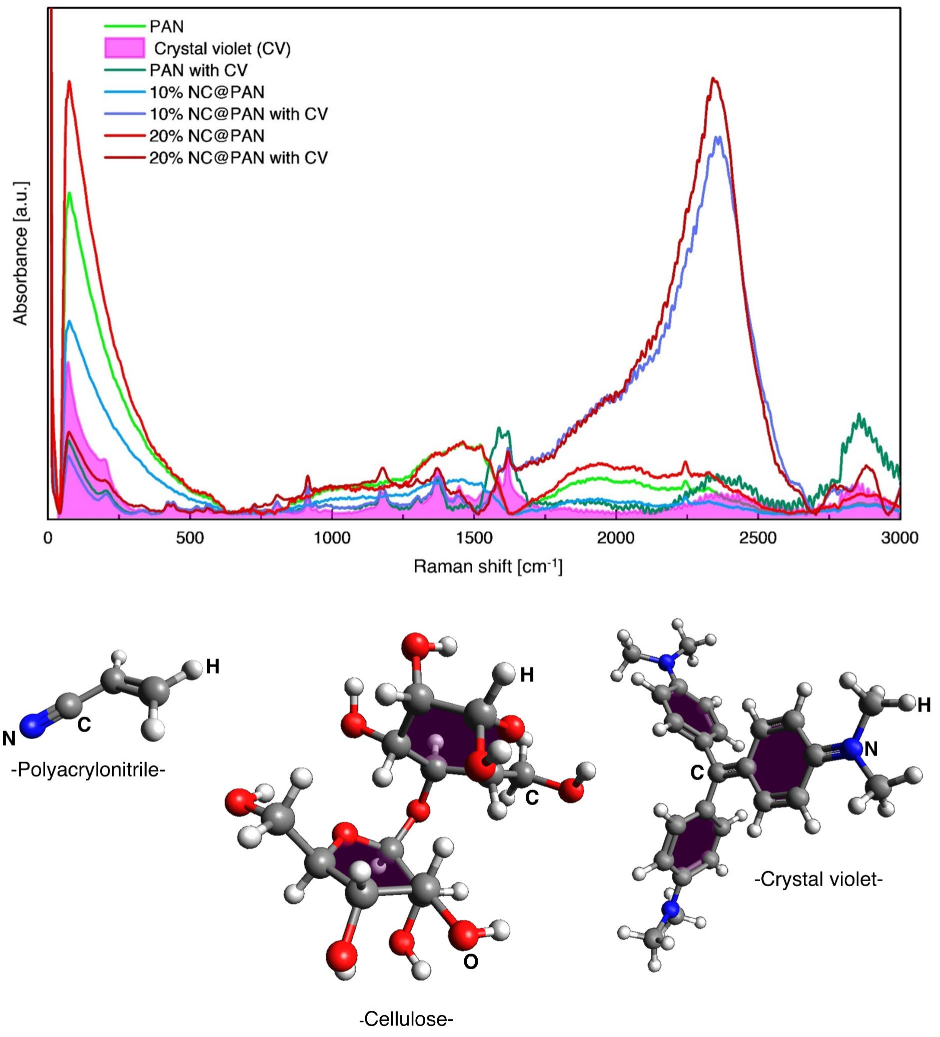

Figure S5. *Micro-Raman survey spectra of pure crystal violet; PAN, 10% NC@PAN, 20% NC@PAN before and after adsorption. Peak smoothing was performed using the adjacent averaging method with a window size of 6 and a polynomial order of 2. Molecular structures of PAN, NC and CV are also displayed.*

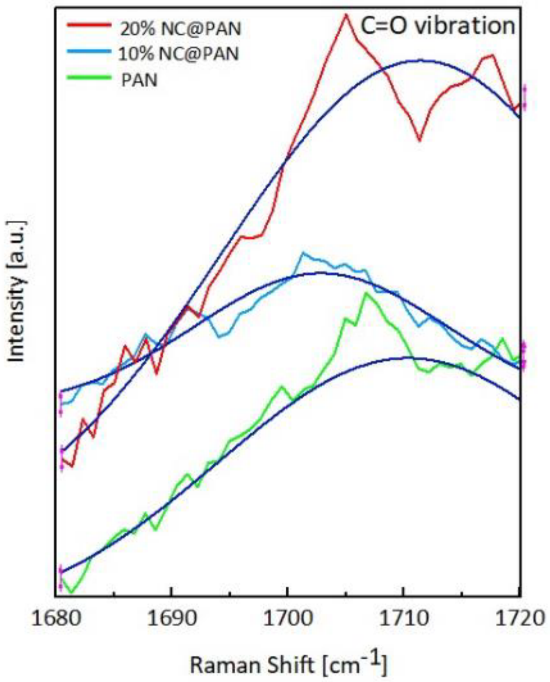

Figure S6. *Confocal micro-Raman spectroscopy of PAN, 10% NC@PAN, 20% NC@PAN before adsorption of CV. Cumulative Gauss fit (blue solid line) and corresponding fitted peaks were selected based on the best R^2. PAN (R^2 : 0.90); 10% NC@PAN (R^2 : 0.90); 20% NC@PAN (R^2 : 0.94).*

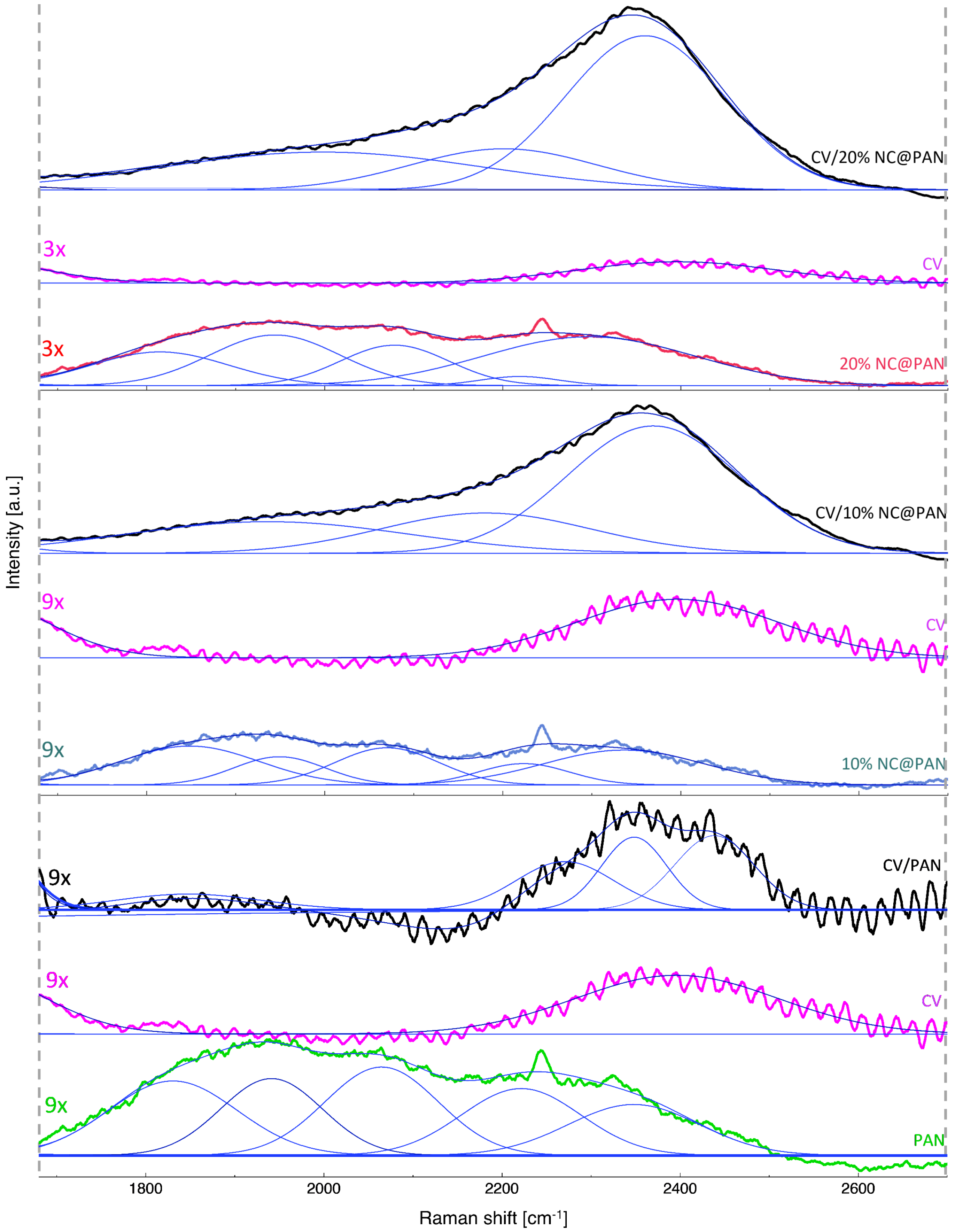

Figure S7. Confocal micro-Raman spectroscopy of pure crystal violet; PAN, 10% NC@PAN, 20% NC@PAN before and after adsorption. Cumulative Gauss fits (blue solid line) and corresponding deconvoluted peaks were selected based on the best R^2. PAN (R^2 : 0.97); crystal violet (R^2 : 0.93); PAN with dye (R^2 : 0.96); 10% NC@PAN (R^2 : 0.98); 10% NC@PAN with dye (R^2 : 0.99); 20% NC@PAN (R^2 : 0.97); 20% NC@PAN (R^2 : 0.99).

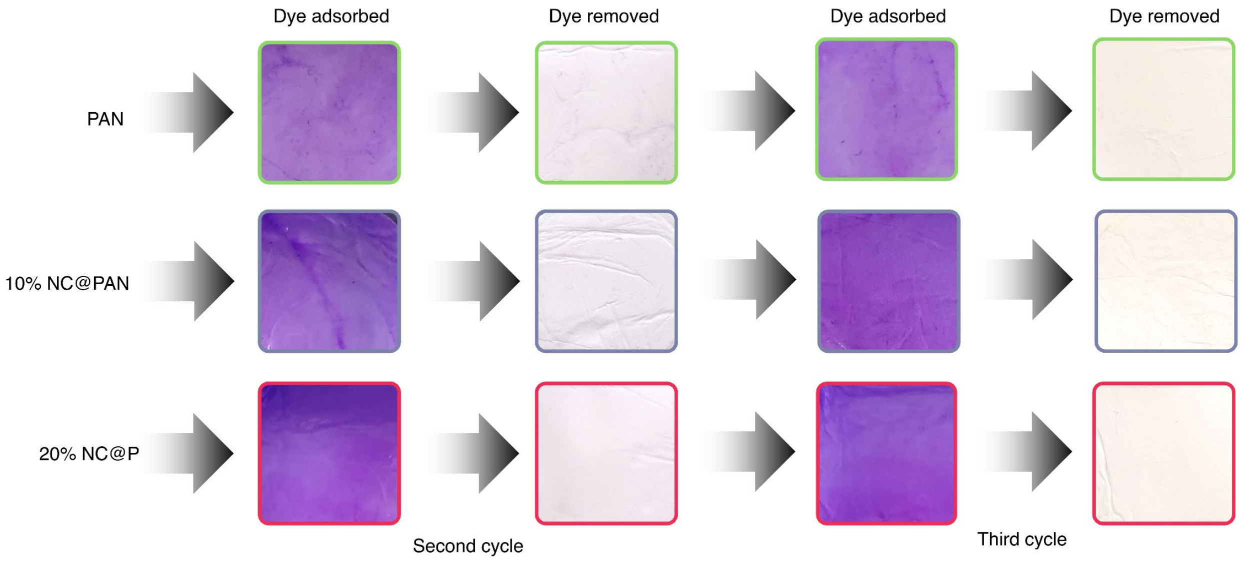

Figure S8. Adsorbed-CV desorption by 1% NaOH treatment, in second and third cycle (decolorization of the nanowebs due to CV removal).

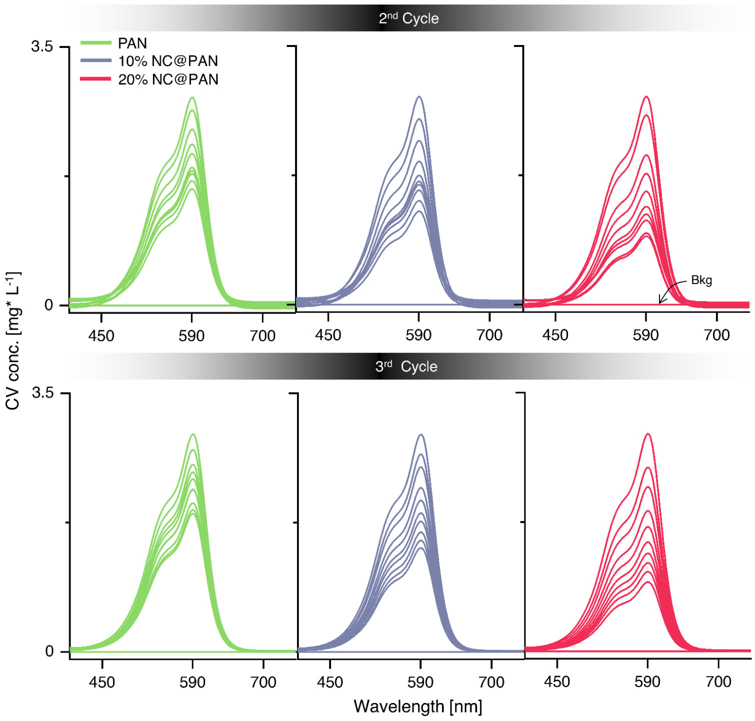

Figure S9. Change in CV concentration (second and third cycle) due to adsorption monitored at abs_{max}@590 nm up to 90 min. by UV spectrophotometry.

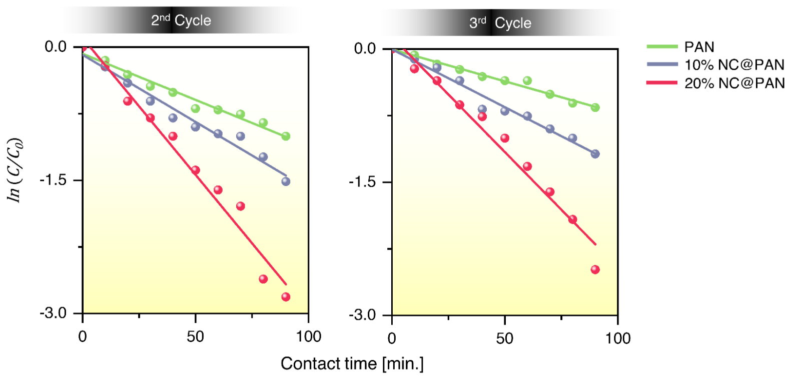

Figure S10. Change in CV concentration $In(C/C_0)$ over time (second and third cycle) by UV spectrophotometry.

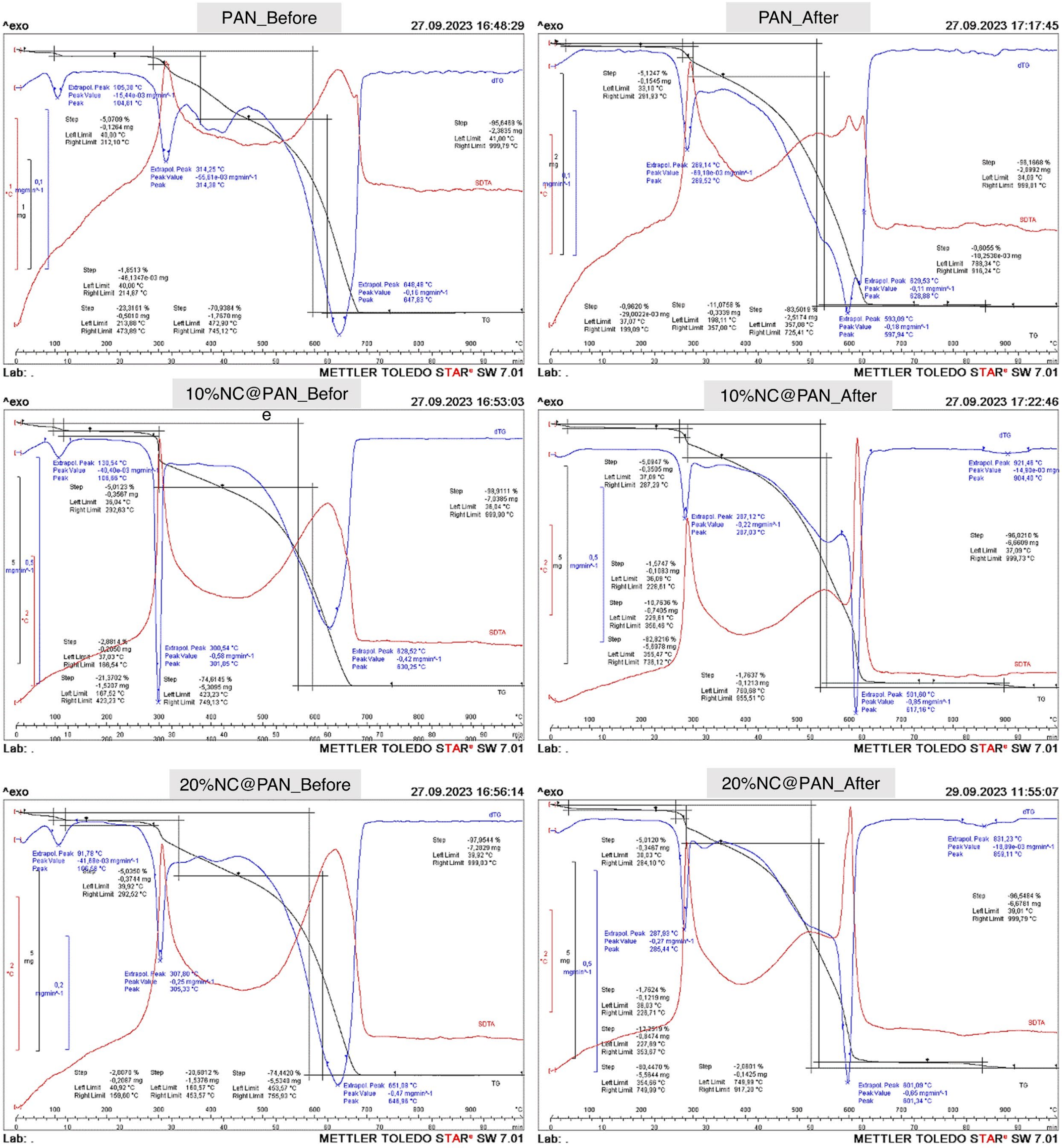

Figure S11. TG, DTG and SDTA curves of PAN nanowebs with 0, 10%, 20% NC before and after exposure to CV.

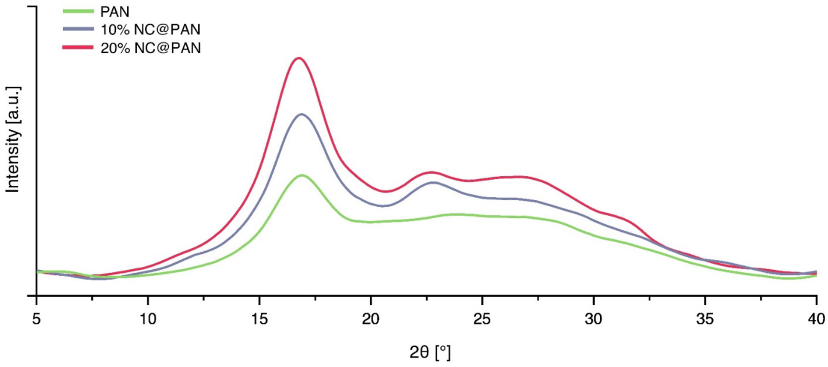

Figure S12. XRD of nanowebs after 3 CV adsorption cycles.

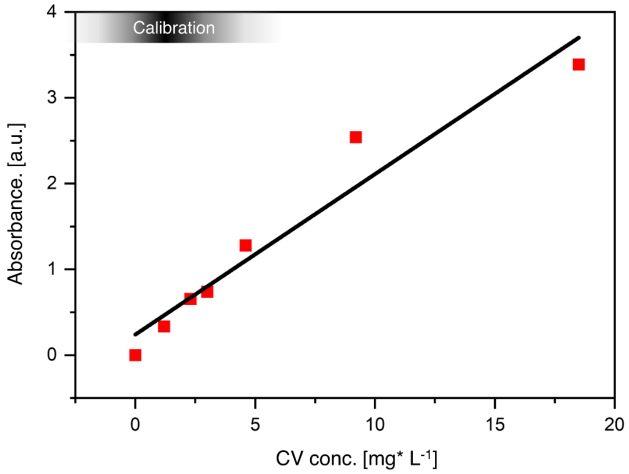

Figure S13. UV-Vis spectroscopy calibration: absorbance intensity vs. CV concentration.

Supplementary note S1

i- Weight calculations for PAN

Density of DMF= 0.948 g/mL
10.5% PAN in DMF –> 5.25 g PAN in 44.75 g DMF
44.75 g / 0.948 g/mL = 47.2 mL DMF
47.2 mL of DMF: 50 g (total)

4 mL (volume of PAN solution used for electrospinning) : x
X = 4.24 g (total weight of the solution)
5.25 g PAN : 50 g (total)
X : 4.24 g
X = 0.44 g PAN weight in the 4 mL sample

18 cm × 18 cm = 324 cm^2 (total area of flat plate collector, collecting nanowebs)
6.5 cm × 6.5 cm = 42.25 cm^2 (used area of nanowebs)
0.44 g PAN :324 cm^2
X : 42.25 cm^2

X = <u>0.057 g PAN</u> weight used for CV adsorption

ii- Weight calculations for 10% NC@PAN

Density of DMSO 1.1 g/mL
Density of DMF 0.948 g/mL
1.25 g nanocellulose + 41 ml DMSO
45.1 g + 1.25 g = 46.35 g (nanocellulose + DMSO)
Weight of DMSO = 41 ml ×1.1 g/ml = 45.1 g

10 ml PAN + 1 mL nanocellulose
5.25g PAN in 44.75 g DMF (47.2 ml)
5.25 g PAN : 47.2 mL
X : 10 ml
X = 1.11 g PAN

1.25 g nanocellulose in 45.1 g DMSO (41 mL)
1.25 g nanocellulose : 41 mL
X :1 mL
X = 0.03 g nanocellulose

10 ml PAN + 1 mL nanocellulose combined
1.11 g PAN : 11 mL
X : 4 mL
X = 0.4 g PAN

0.4 g PAN : 324 cm^2
X : 42.25 cm^2
X = 0.052 g PAN

0.03 g nanocellulose : 11 mL
X : 4 mL

X = 0.01 g nanocellulose

g nanocellulose : 324 cm^2
x : 42.25 cm^2
x = 0.001 g nanocellulose

0.052 g PAN + 0.001 g nanocellulose = <u>0.053 g of 10% NC@PAN</u> used for CV adsorption

iii- Weight calculations for 20% NC@PAN

10 ml PAN + 2 mL nanocellulose
1.11 g PAN
0.03 g nanocellulose ×2 = 0.06 g
1.11 g PAN : 12 mL
X : 4 mL
X = 0.37 g PAN

0.37 g PAN : 324 cm^2
X : 42.25 cm^2
X = 0.048 g

0.06 g nanocellulose : 12 mL
X : 4 mL
X = 0.02 g nanocellulose

g nanocellulose : 324 cm^2
x : 42.25 cm^2
x = 0.0026 g nanocellulose

0.048 g PAN + 0.0026 g nanocellulose = <u>0.0506 g of 20% NC@PAN</u> used for CV adsorption

Table S1. Kinetic parameters of pseudo-second order and Elovich kinetic models.

Pseudo-second order kinetics model									
**Specimen**	**Linear correlation (R^2)**			q_e ($mg\,g^{-1}$)			K_s ($g\,mg^{-1}\,min.^{-1}$)		
	1st cycle	2nd cycle	3rd cycle	1st cycle	2nd cycle	3rd cycle	1st cycle	2nd cycle	3rd cycle
PAN	0.982	0.986	0.988	1.9202	1.8197	2.1651	1.4204	1.2118	2.4056
10% NC @PAN	0.943	0.973	0.974	1.4632	1.6343	1.8135	0.3770	0.6877	0.8905
20% NC@PAN	0.938	0.959	0.952	1.4198	1.2493	1.3680	0.3263	0.2217	0.2835
**Elovich kinetics model**									
	**Linear correlation (R^2)**			α ($g\,g^{-1}\,min.^{-1}$)			β ($g\,mg^{-1}$)		
	1st cycle	2nd cycle	3rd cycle	1st cycle	2nd cycle	3rd cycle	1st cycle	2nd cycle	3rd cycle
PAN	0.968	0.957	0.983	0.0035	0.0039	0.0030	0.6210	0.6249	0.6070
10% NC @PAN	0.971	0.954	0.994	0.0063	0.0050	0.0054	0.6036	0.6174	0.5839
20% NC@PAN	0.969	0.967	0.995	0.0066	0.0081	0.0079	0.6011	0.5993	0.5828

Table S2. Dye removal performance of cellulose based adsorbents from literature.

Material description	Organic pollutant targeted	Max. removal efficiency	Working pH	Ref.
Nanocellulose/chitosan/PHA	Congo red	75.8%	7	[1]
Lignocellulose-g-poly(acrylic acid)/montmorillonite hydrogel	Methylene blue	83.40	10	[2]
Polyacrylamide/cellulose nanocrystal	Methylene blue	90%	5	[3]
Carboxymethylcellulose/alginate/PVA/rice husk	Direct orange, Direct blue, Direct red, Congo red	80-90%	12	[4]
Synthetic carboxymethyl cellulose-acrylic acid	Methyl orange	84.2%	-	[5]
Cellulose/Graphene Oxide Composite	Methylene blue	89%	10.5	[6]
Cellulose/polyaniline nanocomposite	RBBR RO, RV, RBK	82%	3	[7]
Bentonite/carboxymethyl cellulose-g-poly(2-(dimethylamino) ethyl methacrylate)	Congo red, Methyl orange	87%	7	[8]
Carboxylated cellulose derivative	Auramine-O, Safranin-T	44-78%	4.5-7	[9]
Cellulose hydrogel	Direct Blue 86	35.77%	2	[10]
Nanocrystalline cellulose–chitosan hydrogel	Crystal Violet	95.5%	9	[11]
Cellulose Nanocrystal/Poly Acrylic Acid Nanocomposite	Methylene blue	86.3%	7	[12]
PAN	Crystal Violet	59%	7	This study
10% NC@PAN	Crystal Violet	87.3%	7	This study
20% NC@PAN	Crystal Violet	94%	7	This study

Supplementary references

1. Soon, C. Y. *et al.* Electrospun biocomposite: *J. Mater. Res. Technol.* **8**, 5091–5102 (2019).
2. Shi, Y., Xue, Z., Wang, X., Wang, L. & Wang, A. *Polym. Bull.* **70**, 1163–1179 (2013).
3. Zhou, C., Wu, Q., Lei, T. & Negulescu, I. I. *Chem. Eng. J.* **251**, 17–24 (2014).
4. Bhatti, H. N. *et al. Int. J. Biol. Macromol.* **150**, 861–870 (2020).
5. Zhang, G., Yi, L., Deng, H. & Sun, P. *J. Environ. Sci.* **26**, 1203–1211 (2014).
6. Shi, H., Li, W., Zhong, L. & Xu, C. *Ind. Eng. Chem. Res.* **53**, 1108–1118 (2014).
7. Janaki, V., Vijayaraghavan, K., Oh, B. T., Ramasamy, A. K. & Kamala-Kannan, S. *Cellulose* **20**, 1153–1166 (2013).
8. Li, W. *et al. Chem. Eng. Res. Des.* **124**, 260–270 (2017).
9. Teodoro, F. S. *et al. J. Colloid Interface Sci.* **512**, 575–590 (2018).
10. Shoaib, A. G. M., Ragab, S., El Sikaily, A., Yılmaz, M. & El Nemr, A. *Sci. Reports 2023 131* **13**, 1–18 (2023).
11. Poornachandhra, C. *et al. RSC Adv.* **13**, 4757–4774 (2023).
12. Almuslem, A. S., Alnaim, N., Ibrahim, S. S. & Ibrahim, M. A. *Polym. 2023, Vol. 15, Page 2154* **15**, 2154 (2023).
